# Identification of Novel Mutations in Chinese Infants With Citrullinemia

**DOI:** 10.3389/fgene.2022.783799

**Published:** 2022-03-03

**Authors:** Zhi Cheng, Xiwen He, Fa Zou, Zhen-E Xu, Chun Li, Hao Liu, Jingkun Miao

**Affiliations:** ^1^ Key Laboratory of Birth Defects and Reproductive Health of the National Health and Family Planning Commission (Chongqing Population and Family Planning Science and Technology Research Institute), Chongqing, China; ^2^ College of Basic Medical Sciences, Chongqing Medical University, Chongqing, China; ^3^ Department of Neonatology, Children’s Hospital of Chongqing Medical University, Chongqing, China; ^4^ National Clinical Research Center for Child Health and Disorders, Ministry of Education Key Laboratory of Child Development and Disorders, Chongqing Key Laboratory of Pediatrics, Chongqing, China; ^5^ Neonatal Disease Screening Center, Chongqing Health Center for Women and Children, Chongqing, China

**Keywords:** citrullinemia, genetic analysis, mutation, ASS1, SLC25A13

## Abstract

Citrullinemia is a rare autosomal recessive disorder characterized by elevated concentrations of citrulline in the blood resulting from malfunction of the urea cycle. It is categorized into two types, types I and II, which are caused by argininosuccinate synthase 1 (*ASS1*), and citrin (*SLC25A13*) gene mutations, respectively. In this study, we performed genetic analysis on nine Chinese infants with citrullinemia using next-generation sequencing, which identified a novel mutation (p.Leu313Met) and a rare mutation (p.Thr323Ile, rs1250895424) of *ASS1*. We also found a novel splicing mutation of *SLC25A13*: c.1311 + 4_+7del. Functional analysis of the *ASS1* missense mutations showed that both significantly impaired the enzyme activity of ASS1, with the p. Thr323Ile mutation clearly affecting the interaction between ASS1 and protein arginine methyltransferase 7 (PRMT7). These findings expand the mutational spectrum of *ASS1* and *SLC25A13*, and further our understanding of the molecular genetic mechanism of citrullinemia in the Chinese population.

## Introduction

Citrullinemia is a rare autosomal recessive disorder characterized by elevated concentrations of citrulline in the blood from malfunctions of the urea cycle (Saheki et al., 1987). It is categorized into two types according to the molecular pathogenesis. Type I citrullinemia (CTLN1, OMIM# 2,15,700) is caused by argininosuccinate synthase 1 gene (*ASS1*) mutations, while type II citrullinemia is caused by citrin gene (*SLC25A13*) mutations (Woo et al., 2014).

Classic CTLN1 often presents early in the neonatal period in affected individuals with acute hyperammonemia and neurologic manifestations. If untreated, it can lead to life-threatening encephalopathy, metabolic coma, and death (Häberle et al., 2002). Late-onset forms of CTLN1 can also occur. These usually have milder phenotypes, including neurodisability, somnolence, and chronic intermittent hyperammonemia during childhood and adulthood (Häberle et al., 2003).

Type II citrullinemia has two main clinical phenotypes: neonatal intrahepatic cholestatic hepatitis caused by citrin deficiency (NICCD; OMIM# 6,05,814) and adult-onset type II citrullinemia (CTLN2; OMIM# 6,03,471) (Saheki and Kobayashi, 2002). NICCD is clinically characterized by intrahepatic cholestasis and metabolic abnormalities including multiple aminoacidemia, galactosemia, hypoglycemia, and hypoproteinemia. Most patients improve spontaneously without medical treatment before 1 year of age. However, some develop severe CTLN2 one or more decades later (Liu et al., 2014). Patients with CTLN2 suffer from various neuropsychological symptoms including disorientation, delirium, seizures, and coma because of hyperammonemia. Death from brain edema occurs in some cases (Tabata et al., 2008).

*ASS1* is located at chromosome 9q24.11–9q23.12 and contains 16 exons. It encodes the argininosuccinate synthetase enzyme, which catalyzes the synthesis of argininosuccinate from citrulline, and aspartate. It is mainly expressed in the periportal hepatocytes of the liver, but also in most other body tissues (Engel et al., 2009). At least 153 *ASS1* CTLN1 disease-causing mutations have been reported, of which most are missense mutations distributed within exons 3–15 (Diez-Fernandez et al., 2017).

*SLC25A13* is located at chromosome 7q21.3. It encodes citrin, which functions as a calcium (Ca^2+^)-stimulated aspartate-glutamate carrier. Citrin is expressed in many tissues but most abundantly in the liver, and is localized to the mitochondrial inner membrane (Iijima et al., 2001). The first *SLC25A13* disease-causing mutation was identified in a Japanese family with CTLN2 (Kobayashi et al., 1999). Later, some NICCD patients were also shown to carry homozygous and compound heterozygous mutations of *SLC25A13* (Yamaguchi et al., 2002). To date, more than 110 pathogenic mutations of *SLC25A13* have been reported, of which most are point mutations or short insertions/deletions (InDels). These were mainly identified in east Asian populations, including Japanese, Korean, and Chinese (Zhang et al., 2017).

There are currently no well-recognized clinical/biochemical diagnostic criteria for either type of citrullinemia, yet molecular genetic analysis is critical for the diagnosis of patients. In this study, we performed genetic analysis of Chinese infants with citrullinemia using next-generation sequencing (NGS). We also carried out a functional investigation of *ASS1* mutations identified in this study to better understand the genetic mechanism of this disease in the Chinese population.

## Materials and Methods

### Subjects

From June 2014 to December 2020, nine infants with citrullinemia were enrolled in this study. The patients were diagnosed based on clinical findings and biochemical characterization. Clinical and biochemical data were recorded. Whole blood samples were collected for genetic analysis. A cohort of 100 healthy men was studied as a control group.

### Genetic Analysis

Genomic DNA was extracted from the whole blood of all recruited subjects using DNA isolation kits (Tiangen, Beijing, China). A total of 3 µg genomic DNA of affected infants was used to prepare indexed Illumina libraries according to the manufacturer’s protocol (Illumina, San Diego, CA, United States). Coding exons and flanking regions of 165 genes reported to be mutated in disorders of amino acid, organic acid, and fatty acid metabolism were selected and captured using the Agilent SureSelect Target Enrichment System (Agilent, Santa Clara, CA, United States). A list of the targeted genes is provided in Supplementary Table S1. The enriched libraries were sequenced on an Illumina HiSeq 2500 sequencer.

After sequencing, low-quality reads and adaptor sequences were filtered out using the Solexa QA package and the cutadapt program (https://cutadapt.readthedocs.org/), respectively (Cox et al., 2010). Clean reads were aligned to the human reference genome (hg19) using the SOAPaligner program (Li et al., 2009), which was also used to identify single nucleotide polymorphisms (SNPs). To detect InDels, reads were realigned to the reference genome using the Burrows-Wheeler alignment tool, and InDels were identified with the Genome Analysis Toolkit (Li and Durbin, 2009; DePristo et al., 2011). The impact of non-synonymous mutations was assessed *in silico* using Polyphen2 and SIFT (Kumar et al., 2009; Adzhubei et al., 2010), while the effect of splice site mutation was predicted by MutationTaster (Schwarz et al., 2014). The novelty of the mutations was confirmed by searching in dbSNP (http://www.ncbi.nlm.nih.gov/snp/), the 1,000 Genomes Project (https://www.internationalgenome.org/), and the HGMD Professional (http://www.hgmd.cf.ac.uk/ac/index.php) databases. The novel mutations were also confirmed by Sanger sequencing. Conservation analysis was performed using CLC Main Workbench Software.

### Plasmid Construction

The open reading frame (ORF) of human *ASS1* was amplified by PCR from cDNA and inserted into the *Bgl* II- and *Bam*H I-digested pEGFP-N1 vector. p. Leu313Met and p. Thr323Ile mutations were introduced into wild-type (WT) expression plasmids by PCR-based site-directed mutagenesis. Then, the ORFs of WT and mutant *ASS1* were amplified and inserted into the *Nde* I- and *Xba* I-digested pCMV5-FLAG vector to create FLAG-tagged expression plasmids. The ORF of human *PRMT7* was also PCR-amplified and inserted into *Nde* I and *Xba* I sites of the pCMV5-FLAG vector to create expression plasmids. All plasmids were verified by sequencing. The sequences of primers used in plasmid construction are shown in Supplementary Table S2.

### Cell Culture and Transient Transfection

Human embryonic kidney cells (HEK 293) were purchased from Shanghai cell bank (Chinese Academy of Sciences) and were cultured in Dulbecco’s modified Eagle medium (DMEM) supplemented with 10% fetal bovine serum, 100 U/ml penicillin, and 100 μg/ml streptomycin in a humidified incubator containing 5% CO_2_ at 37°C. Transient transfection was carried out using Lipofectamine 2000 (Invitrogen, Carlsbad, CA, United States) according to the manufacturer’s instructions.

### ASS1 Immunoprecipitation and Activity Assay

ASS1 immunoprecipitation was performed as described previously with minor modifications (Herrera Sanchez et al., 2017; Miyamoto et al., 2017). Briefly, FLAG-tagged expression plasmids of ASS1 (WT or mutant) and empty vector were transfected into HEK 293 cells. Two days after transfection, cells were collected and lysed in NP-40 lysis buffer (Beyotime, Shanghai, China). The supernatants were collected after centrifugation at 10,000 × *g* for 15 min at 4°C and incubated with anti-FLAG M2 Beads (Sigma-Aldrich, Shanghai, China) at 4°C for 4 h. The beads were then washed three times with lysis buffer. Bound proteins were eluted by adding 100 μg/ml 3 × FLAG peptide, and eluted proteins were used for ASS1 activity assays.

For these assays, equal amounts of eluted proteins were resuspended in reaction buffer (20 mM Tris-HCl, pH 7.8, 2 mM ATP, 2 mM citrulline, 2 mM aspartate, 6 mM MgCl_2_, 20 mM KCl, and 0.1 U pyrophosphatase) to a final volume of 100 µl. Samples were incubated for 30 min at 37°C, then the reactions were stopped by the addition of 100 µl molybdate buffer (10 mM ascorbic acid, 2.5 mM ammonium molybdate, and 2% sulfuric acid). The accumulation of pyrophosphate was determined at 660 nm by spectrophotometry. Experiments were performed in triplicate and repeated three times.

### Co-Immunoprecipitation and Western Blotting

Expression plasmid pCMV5-FLAG-PRMT7 was co-transfected with pEGFP-ASS1, pEGFP-ASS1-Leu313Met, pEGFP-ASS1-Thr323Ile, or pEGFP-N1 empty vector into HEK293 cells. Immunoprecipitation was carried out as described above. For western blotting, cell lysates and immunoprecipitates were separated by sodium dodecyl sulfate polyacrylamide gel electrophoresis and transferred to nitrocellulose membranes. The membranes were blocked overnight with 5% (w/v) non-fat milk in Tris-buffered saline with 0.1% Tween 20, then probed with anti-green fluorescent protein (GFP) (Sungene, Tianjin, China), anti-β-actin (Sungene), or anti-FLAG M2 (Sigma-Aldrich, Shanghai, China) primary antibodies. They were then incubated with horseradish peroxidase-conjugated goat anti-mouse or goat anti-rabbit secondary antibodies and visualized by enhanced chemiluminescence (Sigma-Aldrich).

## Results

### Patient Characteristics

A total of nine patients (three females, six males) were included in this study. All had high plasma citrulline levels (>1,00 μmol/L), and most had elevated levels of blood arginine, methionine, and threonine. Five patients presented with hyperammonemia. Patient clinical and biochemical characteristics are summarized in Table 1.

**TABLE 1:** Clinical and Biochemical characteristics of patients.

Patients ID	Gender	Age of onset	Blood ammonia (9–33 μmol/l)^*^	Initial plasma amino acids (μmol/l)						
				Arginine (1.5–25)^*^	Citrulline (7–40)^*^	Methionine (8–35)^*^	Serine (20–100)^*^	Threonine (15–100)^*^	Tyrosine (20–100)^*^	Ornithine (15–80)^*^
P1	Male	18 months	60	89.1	416.9	58.6	32.3	119.9	168.3	39.6
P2	Male	1 month	29	63.2	248.9	312.6	58.0	233.9	283.1	58.5
P3	Male	12 months	26	19.5	115.6	37.2	46.7	80.3	93.8	33.7
P4	Female	2 months	39.8	29.6	100.4	21.9	21.7	71.5	31.3	36.0
P5	Female	1 month	10	24.2	359.1	178.1	21.2	142.6	107.6	48.9
P6	Male	3 months	60	78.8	171.9	41.9	19.6	95.8	326.0	29.9
P7	Male	2 months	43.2	58.1	331.2	49.7	54.1	130.2	125.5	134.7
P8	Male	1 month	119.7	119.7	671.3	40.4	26.2	173.7	52.2	48.2
P9	Female	1 month	23.3	23.3	1924.1	51.8	25.0	16.8	38.3	39.3

The symbol “*” indicates reference value.

### Mutational Spectrum

Six mutations (15 mutated alleles) of *SLC25A13* were identified in the patients through NGS. The most common was c.851_854del (seven alleles, 47%), followed by c.1638_1660dup23 (four alleles, 27%). A novel splicing mutation of *SLC25A13* was identified: c.1311 + 4_+7del (Supplementary Figure S1), which resulted in a deletion of “AGUA” at the 5′ splice site. This mutation was not observed in healthy controls, nor reported in dbSNP, 1,000 Genome Project, or the HGMD Professional databases. It was predicted to result in splice site changes and be disease-causing by MutationTaster.

**FIGURE 1:**
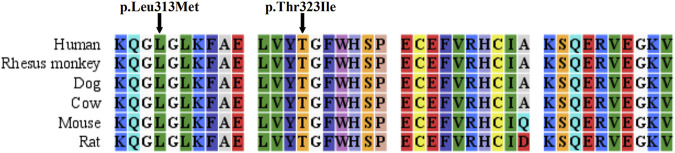
Sequence alignment of ASS1 proteins among species.

Three *ASS1* mutations were detected in the patients, of which a missense mutation was novel: c.937C > A (p.Leu313Met, shown in Supplementary Figure S2). The c.968C > T (p.Thr323Ile) mutation (rs1250895424, shown in Supplementary Figure S3) was identified by Trans-Omics for Precision Medicine (TOPMed) program previously. The minor allele frequency (MAF) is 0.000008. In the Genome Aggregation Database (gnomAD), the MAF of this mutation is 0.00002 in Asian population. So it was a rare mutation. Both mutations were not detected in healthy controls. And both are located at sites that are highly conserved among species (Figure 1). They were predicted to be pathogenic by Polyphen2 and SIFT (Supplementary Table S3). The mutations identified in this study are summarized in Table 2.

**TABLE 2:** Mutations detected in included infants.

	Mutations	Gene	Location	rsID	Type	Patients ID
Homozygotes (*n* = 3)	c.851_854del (p.Met285Profs*2)	*SLC25A13*	Exon 9	rs80338720	Frameshift	P1
	c.851_854del (p.Met285Profs*2)	*SLC25A13*	Exon 9	rs80338720	Frameshift	P2
	c.1638_1660dup23 (p.Ala554Glyfs*17)	*SLC25A13*	Exon 16	rs80338725	Frameshift	P3
Compound heterozygotes (*n* = 6)	c.615+5G > A (p.Ala206Valfs*7)	*SLC25A13*	Intron 6	rs80338717	Frameshift	P4
	c.640C > T (p.Gln214*)		Exon 7	—	Nonsense	
	c.851_854del (p.Met285Profs*2)	*SLC25A13*	Exon 9	rs80338720	Frameshift	P5
	c.1638_1660dup23 (p.Ala554Glyfs*17)		Exon 16	rs80338725	Frameshift	
	c.851_854del (p.Met285Profs*2)	*SLC25A13*	Exon 9	rs80338720	Frameshift	P6
	c.1638_1660dup23 (p.Ala554Glyfs*17)		Exon 16	rs80338725	Frameshift	
	c.1311 + 4_+7del^§^	*SLC25A13*	Intron 13	—	Splicing	P7
	c.1762C > T (p.Arg588*)		Exon 17	—	Nonsense	
	c.851_854del (p.Met285Profs*2)	*SLC25A13*	Exon 9	rs80338720	Frameshift	P8
	c.968C > T (p.Thr323Ile)	*ASS1*	Exon 13	rs1250895424	Missense	
	c.937C > A (p.Leu313Met)^§^	*ASS1*	Exon 13	—	Missense	P9
	c.970+5G > A		Intron 13	—	Splicing	

The symbol “§” indicates novel mutation. And the symbol “–” indicates no record. NM_014251.3 and NM_000050.4 were used as reference sequences for *SLC25A13* and *ASS1*, respectively. The italic values mean the numbers of homozygotes or heterozygotes.

### Functional Analyses of Missense Mutations

**FIGURE 2:**
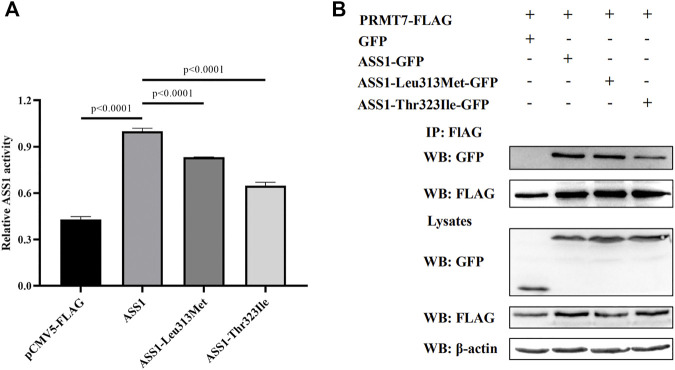
Functional analysis of *ASS1* mutations. **(A)** Effect of p. Leu313Met and p. Thr323Ile mutations on ASS1 activity. The enzymatic activity of ASS1 immunoprecipitated from HEK293 cells expressing pCMV5-FLAG empty vector, ASS1-FLAG, ASS1-Leu313Met-FLAG, or ASS1-Thr323Ile-FALG was determined. Data are normalized to ASS1 wild-type protein (lane 2). Three independent experiments were performed. Statistical significance was determined by one-way ANOVA. **(B)** Effect of p. Leu313Met and p. Thr323Ile mutations on the ASS1–PRMT7 interaction. HEK293 cells co-expressing PRMT7-FLAG and GFP, or PRMT7-FLAG with ASS1-GFP, ASS1-Leu313Met-GFP, or ASS1-Thr323Ile-GFP were harvested. PRMT7-FLAG was immunoprecipitated using anti-FLAG M2 Beads. Immunoprecipitates were analyzed by western blotting using anti-GFP and anti-FLAG M2 antibodies. Total cell lysates were analyzed by western blotting using anti-GFP, anti-FLAG M2, and anti-β-actin antibodies.

Because splicing mutation can cause improper intron removal and alterations of the ORF, functional analyses were performed of the two *ASS1* missense mutations. WT and mutant ASS1 proteins were over-expressed and purified from HEK293 cells. Assays of enzyme activity showed that both p. Leu313Met and p. Thr323Ile mutant proteins had significantly decreased activity compared with WT (Figure 2A).

PRMT7 was previously reported to interact with ASS1 (Verma et al., 2017), so we next investigated the effect of the mutations on this interaction. Co-immunoprecipitation experiments using FLAG-tagged PRMT7 and GFP-tagged ASS1 revealed similar expression levels of the two mutant proteins with that of WT ASS1 in HEK293 cells. A similar amount of p. Leu313Met mutant protein to WT ASS1 was co-immunoprecipitated by PRMT7. However, less p. Thr323Ile mutant protein was co-immunoprecipitated by PRMT7 compared with WT (Figure 2B), suggesting that the p. Thr323Ile mutant protein binds weakly to PRMT7.

## Discussion

Although citrullinemia types I and II are caused by mutations in different genes, they exhibit partial similarities in clinical phenotypes. In the present study, we performed genetic analysis of nine Chinese infants with citrullinemia, and identified homozygous or compound heterozygous mutations of *ASS1* and *SLC25A13* (Table 2). This demonstrated that genetic analysis can help determine the subgroup of citrullinemia besides clinical and biochemical examinations.

Our study identified a novel splicing mutation of *SLC25A13,* which leads to the deletion of “AGUA” at the 5′ splice site. This mutation is very likely to affect pre-mRNA splicing for three main reasons. First, during the splicing process, the 5′ splice site (CAG/GUAAGU sequence) and 3′ splice site (NYAG/G sequence) are recognized by spliceosome components (Ohno et al., 2018). While most common mutations affect +1 and +2 residues at the 5′ donor splice site and −1 and −2 residues at the 3′ acceptor splice site, any mutations in these canonical sequences could impair the interaction between pre-mRNA and the spliceosome, leading to abnormal pre-mRNA splicing (Anna and Monika, 2018). The *OXCT1* c.1248+5G > A mutation, *IKBKAP* c.2204+6T > C mutation, and *CDHR1* c.2040+5G > T mutation were previously found to cause exon skipping (Ibrahim et al., 2007; Hori et al., 2013; Stingl et al., 2017). Moreover, the *ATF6* c.82+5G > T mutation led to intron retention (Kohl et al., 2015), while *ASS1* mutations c.773+4A > C and c.970+5G > A were identified in patients with citrullinemia (Kobayashi et al., 1995; Lin et al., 2019). Second, the MutationTaster predicted that the mutation was disease-causing. Finally, high levels of plasma citrulline (331.2 μmol/l; normal: 7–40 μmol/l) were detected in the patient carrying this mutation. Considering that he also carried a nonsense mutation of *SLC25A13* (p.Arg588*), it is likely that these compound heterozygous mutations caused the production of defective citrin protein.

We also identified a novel mutation (p.Leu313Met) and a rare mutation (p.Thr323Ile) of *ASS1*, which are both located at highly conserved sites and were absent from the 100 healthy controls. Polyphen2 and SIFT predicted them to be damaging, and functional analyses showed that they significantly impaired the enzyme activity of ASS1. Furthermore, *PRMT7* encodes a protein arginine methyltransferase which catalyzes arginine methylation. A previous study showed that PRMT7 interacts with ASS1, and that several mutations associated with citrullinemia disrupt this interaction (Verma et al., 2017). In this study, we found that the p. Thr323Ile mutation also considerably decreased the binding of ASS1 to PRMT7. Therefore, we believe that these two mutations are pathogenic for citrullinemia.

Digenic inheritance refers to mutations in two distinct genes causing a genetic phenotype or disease. With increasing exome and genome sequence data being generated through NGS, the number of human diseases exhibiting digenic inheritance continues to grow (Gazzo et al., 2016; Deltas, 2018). It provides new insights into the genetics underlying many disorders classically considered monogenic (Schäffer, 2013). Distal renal tubular acidosis (dRTA) is just one example (Nagara et al., 2018). In the present study, we identified a patient (P8) harboring both a heterozygous mutation of *ASS1* (p.Thr323Ile) and a heterozygous mutation of *SLC25A13* (p.Met285Profs*2). The patient had high levels of plasma citrulline (671.3 μmol/l, normal: 7–40 μmol/l) and arginine (119.7 μmol/l, normal: 1.5–25 μmol/l). Together, these results might implicate a putative digenic inheritance mechanism in citrullinemia. A mutated allele of *ASS1* may reduce the formation of argininosuccinate from citrulline and aspartate, while a mutated *SLC25A13* allele is likely to further inhibit the reaction by limiting the supply of aspartate from mitochondria. Thus, the compound mutations would be predicted to lead to the upstream accumulation of citrulline. Further analyses are required to investigate the effects of these compound mutations on cell metabolism.

The clinical presentation of citrullinemia is very heterogeneous. In our study, patient P9 with *ASS1* mutations displayed the most severe clinical symptoms. She had the highest plasma citrulline level (1924.1 μmol/l; normal: 7–40 μmol/l), and died at 1 year of age. This indicates that *ASS1* mutations might cause more severe clinical manifestations than *SLC25A13* mutations. Consistent with this, patient P8 carrying compound heterozygous mutations of *ASS1* and *SLC25A13* also showed more severe clinical manifestations than patients only harboring *SLC25A13* mutations. Most mutations of *SLC25A13* identified in this study led to truncated proteins. However, no firm genotype–phenotype correlations could be observed in patients carrying *SLC25A13* mutations.

The present study had some limitations. First, we did not perform functional analysis of the c.1311 + 4_+7del mutation to support its predicted effect on *SLC25A13* splicing. Second, because of limited sample sources, genetic analysis of the patients’ parents was not carried out to determine if the identified novel mutations were *de novo*; additionally, the segregation test for identified mutations could not be performed.

In summary, we carried out genetic analysis and functional investigation in Chinese infants with citrullinemia. We identified a novel mutation of *ASS1* and a novel mutation of *SLC25A13*. These findings expand the mutational spectrum of *ASS1* and *SLC25A13*, and improve our understanding of the molecular genetic mechanism of citrullinemia in the Chinese population.

## Data Availability

The datasets for this article are not publicly available due to concerns regarding participant/patient anonymity. Requests to access the datasets should be directed to the corresponding author.
