# Supplementary material for: Identification of Novel Mutations in Chinese Infants With Citrullinemia
Source: Front Genet. 2022 Mar 3;13:783799. doi: 10.3389/fgene.2022.783799 (PMC8929347; doi:10.3389/fgene.2022.783799)
Supplement: Supplementary file 1 [file DataSheet1.PDF]

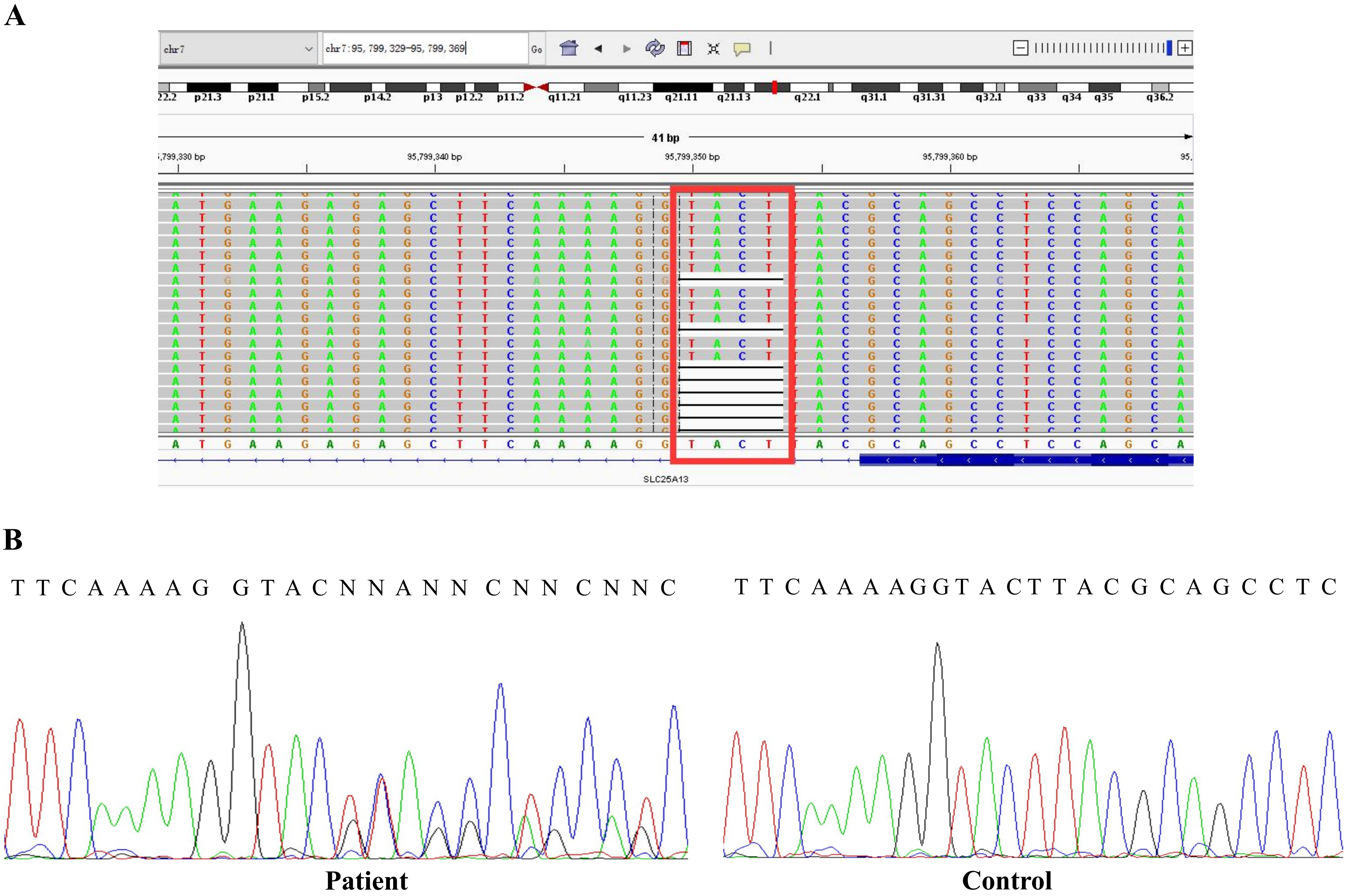

FIGURE S1. The c.1311+4_+7del splicing mutation of *SLC25A13* identified in a patient.
(A) Reads of next-generation sequencing showing the mutation.
(B) Electropherograms obtained by the direct sequencing of PCR products.

A

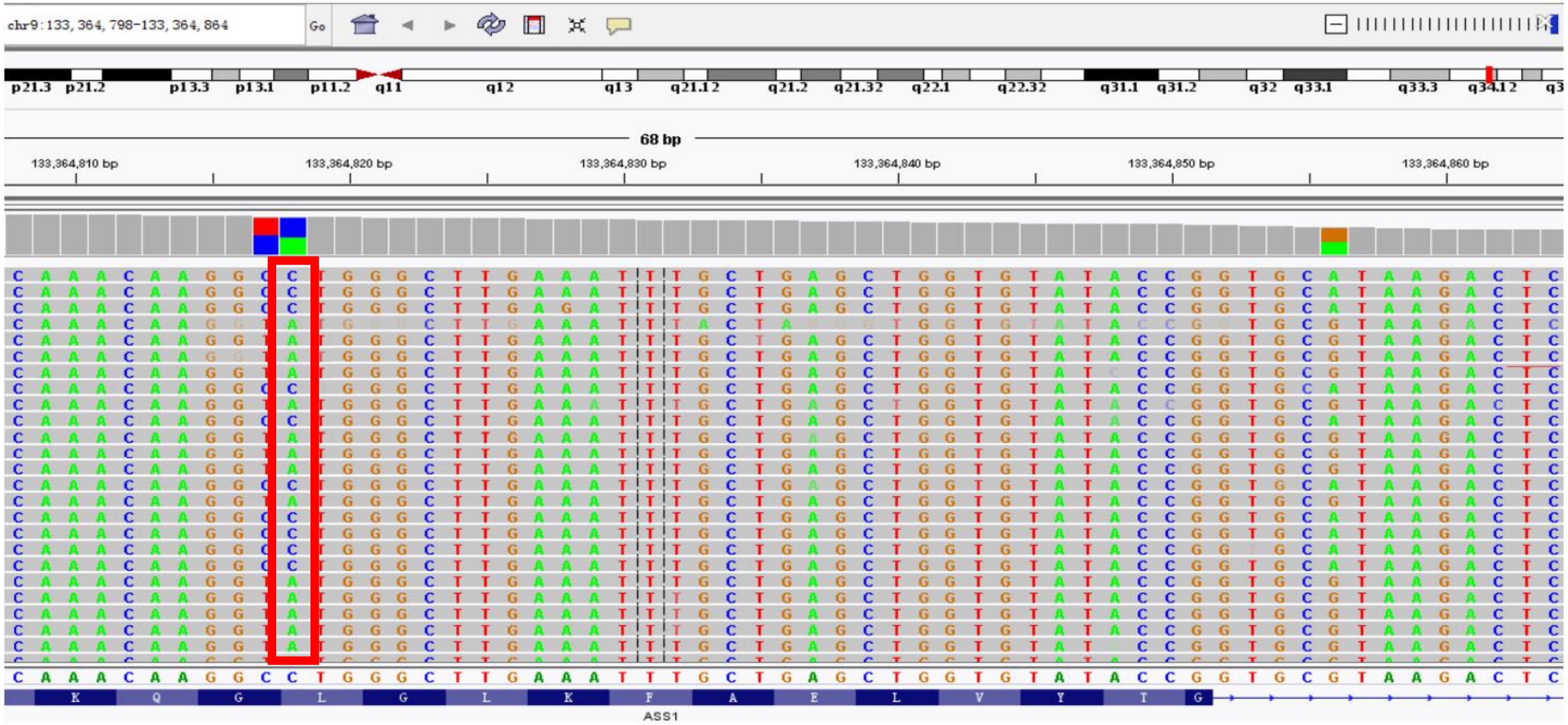

B

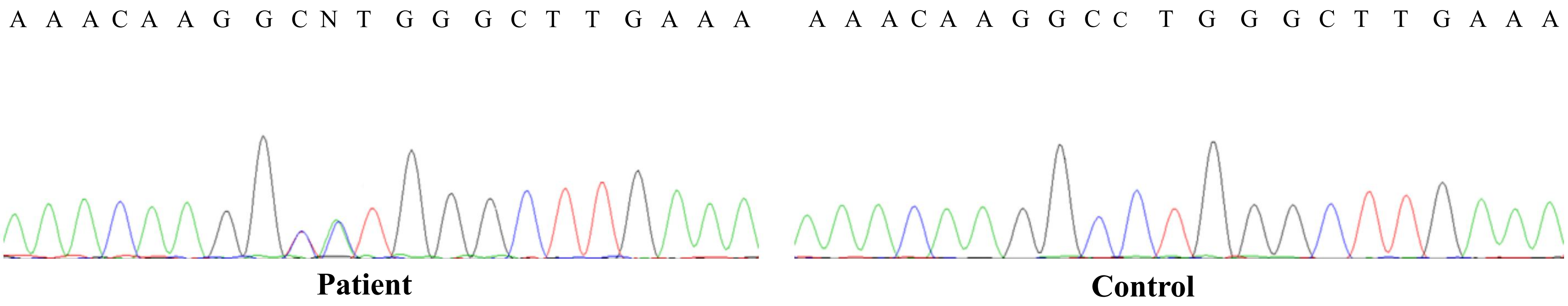

FIGURE S2. The p.Leu313Met mutation of *ASS1* identified in a patient.
(A) Reads of next-generation sequencing showing the mutation.
(B) Electropherograms obtained by the direct sequencing of PCR products.

A

B

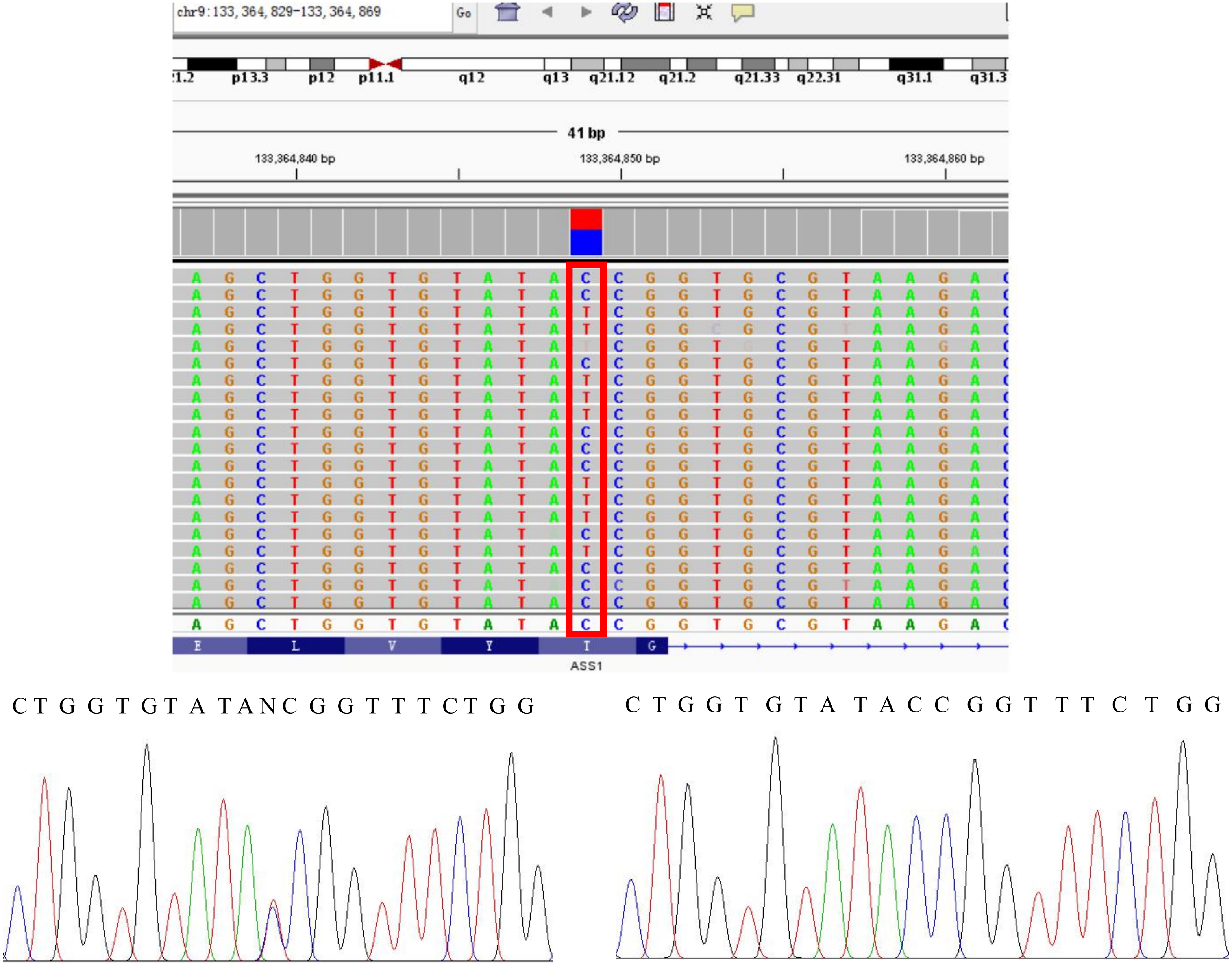

FIGURE S3. The p.Thr323Ile mutation of *ASS1* identified in a patient.

(A) Reads of next-generation sequencing showing the mutation.

(B) Electropherograms obtained by the direct sequencing of PCR products.
