# Supplementary material for: Identification of Novel Mutations in Chinese Infants With Citrullinemia
Source: Front Genet. 2022 Mar 3;13:783799. doi: 10.3389/fgene.2022.783799 (PMC8929347; doi:10.3389/fgene.2022.783799)
Supplement: Supplementary file 2 [file DataSheet2.docx]

**TABLE S1. The genes captured and analyzed in this study**

| AASS | ASL | ETFDH | HADH | MCKAT | PC | SLC3A1 |
| --- | --- | --- | --- | --- | --- | --- |
| ABAT | ASS1 | ETHE1 | HADHA | MCOLN1 | PCBD | SLC46A1 |
| ABCD1 | AUH | FAH | HADHB | MECP2 | PCCA | SLC6A19 |
| ABCD4 | BCAT1 | FH | HAL | MLYCD | PCCB | SLC6A20 |
| ABHD5 | BCAT2 | FOLR1 | HEXA | MMAA | PHGDH | SLC6A8 |
| ACAD8 | BCKDHA | FOLR2 | HEXB | MMAB | PNPLA2 | SLC7A7 |
| ACADM | BCKDHB | FOXG1 | HGD | MMACHC | PNPO | SLC7A9 |
| ACADSB | BTD | FTCD | HLCS | MMADHC | PRODH | SMPD1 |
| ACADVL | SUGCT | G6PC | HMGCL | MOCS1 | PSAP | SPR |
| ACAT1 | CBS | GAA | HPD | MOCS2 | PSAT1 | SUCLG1 |
| ACSF3 | CDKL5 | GALC | HSD17B10 | MTHFR | PSP | SUOX |
| ADK | CPS1 | GALK1 | IDS | MTR | PTS | TAT |
| AGL | CPT1A | GALNS | IDUA | MTRR | QDPR | TAZ |
| AHCY | CPT2 | GAMT | INPP5E | MUT | SARDH | TH |
| ALDH4A1 | CTH | GATM | IVD | MVK | SERAC1 | UROC1 |
| ALDH5A1 | D2HGDH | GBA | L2HGDH | NAGLU | SGSH |  |
| ALDH6A1 | DBH | GCDH | LAMP2 | NAGS | SLC19A1 |  |
| ALDH7A1 | DBT | GCH1 | LMBRD1 | NPC1 | SLC22A5 |  |
| ALPL | DDC | GCSH | MAN2B1 | NPC2 | SLC25A13 |  |
| AMT | DGUOK | GLB1 | MANBA | NTNG1 | SLC25A15 |  |
| APSA | DHFR | GLDC | MAOA | OAT | SLC25A20 |  |
| ARG1 | DHTKD1 | GM2A | MAT1A | OGDH | SLC2A1 |  |
| ARSA | DLD | GNPTAB | MCCC1 | OPA3 | SLC2A2 |  |
| ARSB | ETFA | GNPTG | MCCC2 | OTC | SLC36A2 |  |
| ARX | ETFB | GPHN | MCEE | PAH | SLC37A4 |  |

**TABLE S2. The sequences of primers used in plasmid construction**

|  | **Sequences** |
| --- | --- |
| Primers used to construct pEGFP-ASS1 expression plasmid | Forward: 5’ CTACCGGACTCAGATCTCATGTCCAGCAAAGGCTCC 3’ |
|  | Reverse: 5’ GGCGACCGGTGGATCCCGTTTGGCAGTGACCTTGCTC 3’ |
| Primers used to construct ASS1 p.Leu313Met mutant | Forward: 5’ GCAAAATCAAACAAGGCATGGGCTTGAAATTTGC 3’ |
|  | Reverse: 5’GCAAATTTCAAGCCCATGCCTTGTTTGATTTTGC 3’ |
| Primers used to construct ASS1 p.Thr323Ile mutant | Forward: 5’ CTGAGCTGGTGTATATCGGTTTCTGGCACAG 3’ |
|  | Reverse: 5’ CTGTGCCAGAAACCGATATACACCAGCTCAG 3’ |
| Primers used to construct pCMV5-FLAG-ASS1 expression plasmid | Forward: 5’ GATGACGACAAGCATATGTCCAGCAAAGGCTC 3’ |
|  | Reverse: 5’ CGGGGATCCTCTAGACTATTTGGCAGTGACCTTG 3’ |
| Primers used to construct pCMV5-FLAG-PRMT7 expression plasmid | Forward: 5’ GACGACAAGCATATGATGAAGATCTTCTGCAGTCG 3’ |
|  | Reverse: 5’ CGGGGATCCTCTAGATCAGTCTGGGGTATCTGC 3’ |

**TABLE S3. The effects of ASS1 mutations on protein function by *in silico* analysis**

|  | **Polyphen2** | **SIFT** |
| --- | --- | --- |
| p.Leu313Met | Probably damaging (Score:0.996) | Deleterious (Score: 0.02) |
| p.Thr323Ile | Probably damaging (Score:0.712) | Deleterious (Score: 0) |
